# Fetal Bradycardia Caused by Monogenic Disorders—A Review of the Literature

**DOI:** 10.3390/jcm11236880

**Published:** 2022-11-22

**Authors:** Dominik S. Westphal, Michael Hauser, Britt-Maria Beckmann, Cordula M. Wolf, Gabriele Hessling, Renate Oberhoffer-Fritz, Annette Wacker-Gussmann

**Affiliations:** 1 Department of Internal Medicine I, Klinikum Rechts der Isar, School of Medicine and Health, Technical University Munich, 81675 Munich, Germany; 2 Institute of Human Genetics, Klinikum Rechts der Isar, School of Medicine and Health, Technical University Munich, 81675 Munich, Germany; 3 Praxis für Kinderkardiologie, 80638 Munich, Germany; 4 Institute of Legal Medicine, University Hospital Frankfurt, Goethe University, 60596 Frankfurt, Germany; 5 DZHK (German Centre for Cardiovascular Research), Partner Site Munich Heart Alliance, 80802 Munich, Germany; 6 Department of Congenital Heart Defects and Pediatric Cardiology, German Heart Center Munich, School of Medicine and Health, Technical University Munich, 80636 Munich, Germany; 7 Institute of Preventive Pediatrics, TUM Department of Sport and Health Sciences, Technical University Munich, 80992 Munich, Germany

**Keywords:** fetal, bradycardia, arrhythmia, genetics

## Abstract

**Introduction:** The standard obstetric definition of fetal bradycardia is a sustained fetal heart rate < 110 bpm over at least 10 min. Fetal bradycardia can be the first and only prenatal presentation of a heart disease. We present an overview on different genetic disorders that should be taken into consideration in case of diagnosed fetal bradycardia. **Methods:** A literature review was conducted using a PubMed- and OMIM-based search for monogenetic disorders causing fetal bradycardia in September 2022. **Results:** The review on the literature identified nine monogenic diseases that could lead to fetal bradycardia. Four of these disorders can be associated with extracardiac findings. **Discussion:** Genetic testing should be considered in cases with fetal bradycardia, especially in cases of additional extracardiac findings. Broad sequencing techniques and improved prenatal phenotyping could help to establish a diagnosis in an increasing number of cases.

## 1. Background

In routine obstetrical care, fetal ultrasound, echocardiography (including Doppler techniques) and cardiotocography have been the mainstay of fetal well-being and fetal arrhythmia diagnosis. The American College of Obstetrics and Gynecology defines fetal bradycardia as a fetal heart rate less than 110 beats/min (bpm) [1]. Moreover, sex-specific percentiles are used for heart rate monitoring [2]. A heart rate of <100 bpm in general may be present in 5% of fetuses with arrhythmia [3].

There are several primary causes for fetal bradycardia, such as chromosomal abnormalities [4], but also inherited arrhythmia syndromes caused by ion channel defects, for example [5]. Apart from that, there is a plethora of disorders or circumstances that can lead to secondary fetal bradycardia, e.g., maternal treatment with beta-blockers, SSA/Ro positive pregnancies with heart block [5] and fetal metabolic acidosis [6].

One of the most well-known genetic causes is the channelopathy named Long QT syndrome (LQTS). It was shown that fetal bradycardia is a strong predictor for LQTS [7]. Disease severity is partly influenced by the type of disease-causing variant. It was shown that de novo compared to familial LQTS can present with a more severe prenatal phenotype [8] and that different forms of bradycardia are associated with specific disease genes: sinus bradycardia seemed to be associated with *KCNQ1*, while bradycardia caused by artrioventricular (AV) block was caused by variants in *KCNH2* in neonates in a previous study [9]. Fetal magnetocardiography (fMCG), a non-invasive method to diagnose fetal heart rhythm precisely, proved to be helpful in the prenatal diagnosis of LQTS [10] but is not a widely established method in regular patient care. Due to these limitations, the question becomes in which cases is genetic testing and counseling is helpful during pregnancy or after birth.

In this review we give an overview on different monogenetic diseases that can be causal for fetal bradycardia. The aim is to raise awareness of rare inherited diseases which might not be diagnosed in clinical routine and to emphasize the role of genetic testing.

## 2. Methods

**Figure 1:**
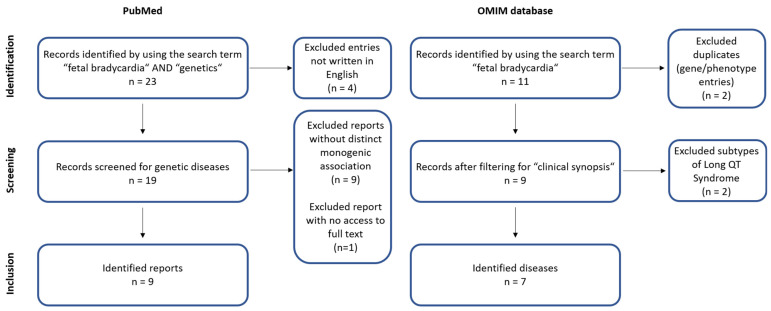
Flowchart depicting the performed OMIM database- and PubMed-based literature search.

In order to study on different monogenic diseases that can be responsible for fetal bradycardia, we performed a review of the literature in September 2022. We conducted an OMIM database-based search (https://omim.org/, accessed on 7 September 2022) for “fetal bradycardia” in the clinical synopses and conducted a literature research in the PubMed database (https://pubmed.ncbi.nlm.nih.gov/, accessed on 7 September 2022) using the key words “fetal bradycardia” AND “genetics”. Publications written in English were screened for clinical cases of fetal bradycardia due to monogenic diseases. Only published studies in humans, including cohort studies and case reports, were considered for the review (Figure 1).

## 3. Results

In order to give an overview on different diseases that can cause fetal bradycardia related to genetic disorders not including chromosomal anomalities, we performed a literature review as described in the methods (overview on identified reports on a PubMed-based search: Table 1). We identified nine different genetic diseases that cause fetal bradycardia published until September of 2022 (summarized in Table 2). One third of the diseases (three out of nine) were inherited cardiac channelopathies (LQTS, Sick Sinus Syndrome and Short QT Syndrome (SQTS)). The other six diseases were systemic disorders affecting different organs or metabolism. One of these entities was tuberous sclerosis, in which the bradycardia was caused by atrioventricular block [11]. Another systemic disorder was Holt-Oram Syndrome (HOS) in which cardiac conduction disease can occur primary without presence of a congenital heart defect (CHD) [12]. In the remaining four disorders, the fetal bradycardia was most likely secondary to the systemic reaction (lethal congenital glycogen storage disease of heart, combined oxidative phosphorylation deficiency, type 41, familial erythrocytosis, type 2 and nuclear mitochondrial complex III deficiency, type 10).

Most of the entities (seven out of nine) presented with further prenatal cardiac and extracardiac symptoms apart from bradycardia. Four of the diseases (44.4%) were associated with extracardiac findings. Only the SQTS and the familial erythrocytosis type 2 have not been associated with further prenatal findings in previous publications, yet. Almost all additional findings were related to arrhythmic or structural cardiac abnormalities. Apart from that, the nuclear mitochondrial complex III deficiency, type 10 was associated with additional intrauterine growth restriction (IUGR) in one reported case [16].

## 4. Discussion

The review of the literature of monogenetic diseases associated with fetal bradycardia identified nine different disorders in this study. One-third of these were cardiac channelopathies (three out of nine). Additionally, in most of the cases (seven out of nine), further prenatal cardiac and extracardiac findings were reported. Especially the appearance of extracardiac findings, which is suggestive for syndromic disorders. However, fetal bradycardia can be the only symptom of a syndromic disease. Variants in *TBX5*, for example, are associated with HOS, classically consisting of upper-limb malformations, CHD and sometimes cardiac conduction disease [12]. Prenatal onset of bradycardia in HOS is not a well-known disease manifestation, but it has been described before in a fetus with ventricular septal defect and pericardial effusion [27]. Cardiac conduction disease, however, was also reported in HOS even without congenital heart disease [12]. Although congenital heart defects and extracardiac findings are indicators for a monogenic disorder, a lack of these finding should not lead to a premature exclusion of a monogenic disorder.

Congenital LQTS is the probably most widely known genetic cause for fetal bradycardia [7]. In case of an identified pathogenic variant of one of the known disease genes, the parents can be counseled regarding the prognosis and possible therapeutic options [22]. However, there are common polymorphisms whose clinical significance can be unclear. For laboratories that are performing genetic sequencing, data interpretation is not trivial when it comes to the interpretation of such polymorphisms. Due to their high frequency in the common population, such variants might be unnoted by automatic variant detections tools depending on the set filters. The identifying and classifying process requires experience and literature-based evaluation. Such polymorphisms cannot be classified solely by the commonly used ACMG criteria [28], which was built for variant interpretation in monogenic diseases. The impact of polymorphisms or genetic disease modifiers, however, will even grow in the future. It was shown for LQTS, for example, that about 15% of disease susceptibility can be explained by polygenic inheritance [29]. This polygenic inheritance is calculated by polygenic risk scores (PRS). PRS were developed for different traits in the meanwhile including resting heart rate [30]. This might help to explain so far unsolved cases that are affected by prenatal arrhythmia, such as fetal bradycardia. Interestingly, congenital LQTS, which is mostly caused variants gene coding for ion channels, is not only restricted to cardiac arrhythmia. Apart from the autosomal dominant LQTS type 1, pathogenic variants in the gene coding for the voltage-gated potassium channel KCNQ1 are associated with additional autosomal recessive inherited congenital sensorineural hearing loss, named Jervell and Lange-Nielsen syndrome [31]. Variants in *CACNA1C* coding for a subunit of a voltage-gated calcium channel are associated with Timothy syndrome, next to the LQTS type 8. Timothy syndrome is a multisystemic disorder with a phenotype spectrum comprising dysmophological features, structural heart defects, skeletal abnormalities and neurological and psychiatric symptoms amongst others [32]. Most symptoms do not manifest prenatally which makes the identification of a disease-causing variant even more significant for the parents’ counselling.

Another channelopathy is the *HCN4*-associated SSS which can lead to a sinus bradycardia onset in utero [33,34]. Our group published in a recent case study that variants in *HCN4* can be associated with further prenatal arrhythmias-like atrial flutter. These additional findings were shown by fetal magnetocardiography [14]. The above mentioned fMCG is a technology which safely and noninvasively records the natural electromagnetic signal of the fetal heart [5,35,36]. FMCG allows precise assessment of cardiac time intervals, signal characteristics, and diverse rhythm patterns. Combined with broad genetic testing and growing knowledge in polygenic inheritance, this improved phenotyping will be a precious tool to characterize and identify complex arrhythmias in the future. The identification of the genetic origin of a fetal arrhythmia is of importance for the further clinical management, such as advising the parents to deliver in a specialized tertiary care center and electrophysiological evaluation by a pediatric cardiologist. Moreover, it can have direct therapeutic consequences, such as avoidance of QT-prolonging drugs in LQTS [22], monitoring the mother and her child during the pregnancy or early treatment with anti-arrhythmic drugs, such as mexiletine in case of a detected ventricular tachycardia by fMCG [37]. The therapeutic relevance of the correct genetic diagnosis might even grow in the future. There are hints, for example, that the alglucosidase-alfa enzyme replacement therapy used for the treatment of patients with Morbus Pompe might also be beneficial for patients with *PRKAG2*-associated glycogen storage disease [38]. Such treatments could be started directly after birth or possibly even in utero in the future. Overlooking a disease-causing genetic variant might not only lead to missed therapeutical treatments, such as the mentioned ones above, but also to an unknown elevated recurrence risk for the parents or even further relatives.

## 5. Conclusions

Fetal bradycardia can be a leading symptom of an inherited genetic disorder and, therefore, genetic testing should be considered, especially in case of additional findings. Broad sequencing in combination with improved prenatal diagnosis will help to establish a genetic arrhythmia diagnosis in a growing number of cases in the near future.

## Figures and Tables

**Table 1:** Identified reports in the PubMed-based database search.

Name	Type	Reference
Case Report: Biventricular Noncompaction Cardiomyopathy With Pulmonary Stenosis and Bradycardia in a Fetus With KCNH2 Mutation	Case report	[13]
The missense variant p.(Gly482Arg) in HCN4 is responsible for fetal tachy-bradycardia syndrome	Case report	[14]
Fetal diagnosis of KCNQ1-variant long QT syndrome using fetal echocardiography and magnetocardiography	Case report	[15]
Bi-Allelic UQCRFS1 Variants Are Associated with Mitochondrial Complex III Deficiency, Cardiomyopathy, and Alopecia Totalis	Case report	[16]
Timothy syndrome 1 genotype without syndactyly and major extracardiac manifestations	Review	[17]
Dysfunctional potassium channel subunit interaction as a novel mechanism of long QT syndrome	Original article	[18]
Prenatal diagnosis of a long QT syndrome by fetal magnetocardiography in an unshielded bedside environment	Case report	[19]
A novel SCN5A mutation manifests as a malignant form of long QT syndrome with perinatal onset of tachycardia/bradycardia	Case report	[20]
Prenatal molecular genetic diagnosis of congenital long QT syndrome by strategic genotyping	Case report	[21]

**Table 2:** Overview on monogenic diseases that can cause fetal bradycardia.

Primary/Secondary Bradycardia	Associated Disease	Gene(s)	Inheritance	Further Prenatal Manifestations
Primary	Long QT Syndrome	*KCNQ1, KCNH2, SCN5A* *	AD, AR	AV block, prolonged QTc [22], syndactyly in Timothy Syndrome [23]
	Sick Sinus Syndrome	*HCN4, SCN5A*	AD, AR	atrial flutter, prolonged QTc [14]
	Short QT Syndrome	*KCNQ1, KCNH2, KCNJ2*	AD	not reported
	Holt Oram Syndrome	*TBX5*	AD	structural heart defects (e.g., VSD), skeletal abnormalities (e.g., upper-limb malformations) [12]
	Tuberous sclerosis	*TSC1, TSC2*	AD	neuronal migration disorder [24], cardiac rhabdomyosarcoma [11]
Secondary	Lethal congenital glycogen storage disease of heart	*PRKAG2*	AD	hypertrophic cardiomyopathy [25]
	Combined oxidative phosphorylation deficiency, type 41	*GATB*	AR	cardiomegaly, fetal hydrops [26]
	Familial erythrocytosis, type 2	*VHL*	AR	not reported
	Nuclear mitochondrial complex III deficiency, type 10	*UQCRFS1*	AR	IUGR [16]

AD: autosomal dominant; AR: autosomal recessive; AV: atrioventricular; IUGR: intrauterine growth restriction. * three most common genes.

## Data Availability

The data that support the findings of this study are available from the corresponding author upon reasonable request.
